# Dexamethasone mediates pancreatic cancer progression by glucocorticoid receptor, TGF*β* and JNK/AP-1

**DOI:** 10.1038/cddis.2017.455

**Published:** 2017-10-05

**Authors:** Li Liu, Ewa Aleksandrowicz, Frank Schönsiegel, Daniel Gröner, Nathalie Bauer, Clifford C Nwaeburu, Zhefu Zhao, Jury Gladkich, Torsten Hoppe-Tichy, Eitan Yefenof, Thilo Hackert, Oliver Strobel, Ingrid Herr

**Affiliations:** 1 Section Surgical Research, Molecular OncoSurgery, University of Heidelberg, Heidelberg, Germany; 2 Department of General Surgery, University of Heidelberg, Heidelberg, Germany; 3 Clinic Pharmacy, University of Heidelberg, Heidelberg, Germany; 4 The Lautenberg Center for Immunology and Cancer Research, IMRIC, Hebrew University–Hadassah Medical School, Jerusalem, Israel

## Abstract

Glucocorticoids such as dexamethasone are widely co-prescribed with cytotoxic therapy because of their proapoptotic effects in lymphoid cancer, reduction of inflammation and edema and additional benefits. Concerns about glucocorticoid-induced therapy resistance, enhanced metastasis and reduced survival of patients are largely not considered. We analyzed dexamethasone-induced tumor progression in three established and one primary human pancreatic ductal adenocarcinoma (PDA) cell lines and in PDA tissue from patients and xenografts by FACS and western blot analysis, immunohistochemistry, MTT and wound assay, colony and spheroid formation, EMSA and *in vivo* tumor growth and metastasis of tumor xenografts on chicken eggs and mice. Dexamethasone in concentrations observed in plasma of patients favored epithelial–mesenchymal transition, self-renewal potential and cancer progression. Ras/JNK signaling, enhanced expression of TGF*β*, vimentin, Notch-1 and SOX-2 and the inhibition of E-cadherin occurred. This was confirmed in patient and xenograft tissue, where dexamethasone induced tumor proliferation, gemcitabine resistance and metastasis. Inhibition of each TGF*β* receptor-I, glucocorticoid receptor or JNK signaling partially reversed the dexamethasone-mediated effects, suggesting a complex signaling network. These data reveal that dexamethasone mediates progression by membrane effects and binding to glucocorticoid receptor.

Pancreatic ductal adenocarcinoma (PDA) is one of the most aggressive malignancies^1^ that frequently arises from chronic pancreatitis, grows embedded in inflamed tissue^2^ and is associated with pronounced cachexia.^3^ Glucocorticoid medication has specific indications in reduction of inflammation,^4, 5^ stimulation of appetite in cancer patients suffering from cachexia,^3^ control of pain and fatigue, suppression of nausea and emesis induced by chemotherapy,^6^ reduction of tumor-related edema in metastatic epidural spinal cord compression^7^ and relief of bowel obstruction.^8^ Glucocorticoids are steroid hormones and cortisol, the major form, is endogenously produced and secreted by the adrenal cortex in response to stress. Synthetic glucocorticoids such as dexamethasone are among the most commonly prescribed anti-inflammatory drugs and their frequent administration in tumor therapy is originally based on their proapoptotic effects in lymphoid malignancies.^6^ However, concerns about the widespread use of glucocorticoids for therapy of solid tumors have been expressed repeatedly,^6, 9, 10^ with studies indicating an increased likelihood of metastasis in breast cancer patients^11, 12^ or increased risks of skin cancer and non-Hodgkin’s lymphoma among users of systemic glucocorticoids.^13^ Several laboratory and animal experiments have demonstrated that glucocorticoids induce resistance to chemotherapy in the majority of solid tumor entities, including PDA.^6, 10, 14^ Glucocorticoids transduce their broad spectrum of activities by binding to its cytoplasmic receptor to form a glucocorticoid receptor complex that is then conveyed to the nucleus under the control of certain chaperones.^15, 16^ In the nucleus, the glucocorticoid receptor binds directly to glucocorticoid response elements in promoter regions^17^ to regulate transcription, or controls the function of other transcription factors, such as NF-*κ*B and AP-1.^18^ This affects the expression of several target genes, one of which is transforming growth factor-*β* (TGF*β*).^16, 19^ In PDA, TGF*β* induces epithelial–mesenchymal transition (EMT),^20^ cancer stem cell (CSC) signaling and metastasis,^21, 22^ involving the upregulation of the mesenchymal marker vimentin and downregulation of the epithelial marker E-cadherin.^23^ TGF*β* autoactivates its own mRNA expression and protein secretion by the induction of the cJun mRNA,^24^ whose protein product is a subunit of the transcription factor AP-1 that in turn transcriptionally regulates the TGF*β* promoter.^25^ Glucocorticoids also modulate multiple kinases such as RAS and JNK, for example, by nonspecific interactions with the cell membrane or binding to a membrane-bound glucocorticoid receptor.^15, 26^ JNK phosphorylates and thereby activates cJun and other AP-1 subunits that regulate the complex program of gene expression that defines an invasive phenotype.^27^

In the present study we show that dexamethasone induces tumor progression of PDA by utilizing a complex signaling network with the major mediators TGF*β*, glucocorticoid receptor and JNK signaling.

## Results

### Dexamethasone favors EMT-related tumor progression

**Figure 1:**
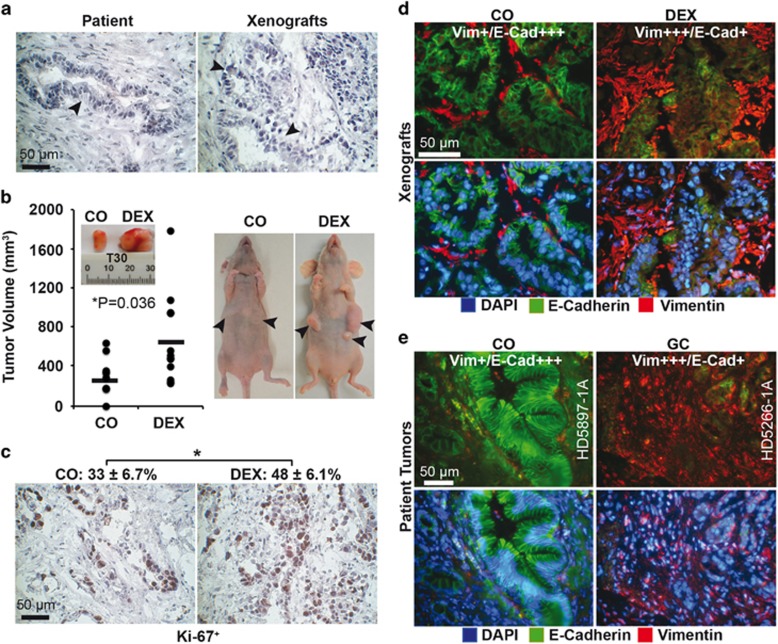
Dexamethasone induces progression ***in vivo*** and ***ex vivo***. (**a**) Immunohistochemistry of a patient PDA tissue section (left) and the corresponding low-passage mouse xenograft (right). The arrows indicate the typical ductal structures of PDA. (**b**) Tumor cells were freshly isolated from the xenograft tissue and cultured as spheroids *in vitro*. One half of the cells were left untreated (CO), and the other half was treated with 1 *μ*M dexamethasone (DEX). After 48 h, equal amounts of viable cells were transplanted onto the left and right flanks of immunodeficient mice (*n*=6). At 3 weeks after transplantation, the xenografts were resected and the volumes determined. Representative photographs of resected tumors and tumor-bearing mice, the individual tumor sizes (black dots) and the means per group (black line) are shown, *P<0.05. (**c**) The expression of the proliferation marker Ki-67 was detected in xenograft sections by immunohistochemistry, under × 400 magnification, and representative images are shown. The percentage of Ki-67-positive cells was determined by counting the number of dark brown cells in 10 visual fields, and the mean percentages±S.D. are shown, *P<0.05. (**d**) Double immunofluorescence staining of xenograft sections for the epithelial marker E-cadherin^29^ and the mesenchymal marker vimentin (red). The cell nuclei were counterstained with DAPI (blue). Cell sections were analyzed under × 400 magnification, and representative images are shown. For evaluation of the expression levels, a semiquantitative scoring system was used, based on the determination of the intensity expression and percentage of positive cells was determined by visual judgment with double-blind method: extremely strong expression and very high percentage (≥75%) (++++), strong expression and high percentage (≥50%) (+++), moderate expression and medium percentage (≥25%) (++), weak expression and low percentage (≤25%) (+) and absent (−). (**e**) Representative paraffin-embedded sections (HD5897-1A, HD5266-1A) derived from primary PDA tumor tissue from patients with documented preoperative administration of glucocorticoids (GC, *n*=17) or not (CO, *n*=17) were evaluated as described above

Cancer spheres were isolated from the primary PDA xenograft line T30 and let either untreated or were treated *in vitro* with 1 *μ*M (0.4 *μ*g/ml) dexamethasone or solvent alone as control. This concentration resembles peak levels of 0.3 to 3 *μ*g/ml that can be measured in patient plasma 2 to 16 h after intake of 2.49 mg/kg dexamethasone.^28^ At 48 h after treatment, the T30 cells were transplanted into the left and right flanks of immunodeficient mice. After 3 weeks, the tumors were resected and examined. The morphology resembled that of the primary patient tumor with the typical ductal structures (Figure 1a). Dexamethasone enhanced the tumor volume and proliferation, as detected by calipers (Figure 1b) and Ki67 staining (Figure 1c), whereas the expression of vimentin increased and the expression of E-cadherin decreased (Figure 1d). The observed expression pattern of vimentin and E-cadherin was confirmed by immunohistochemistry in tissue of PDA patients who had taken glucocorticoids before surgery (*n*=17) or not (*n*=17) (Figure 1e and Table 1).

**Figure 2:**
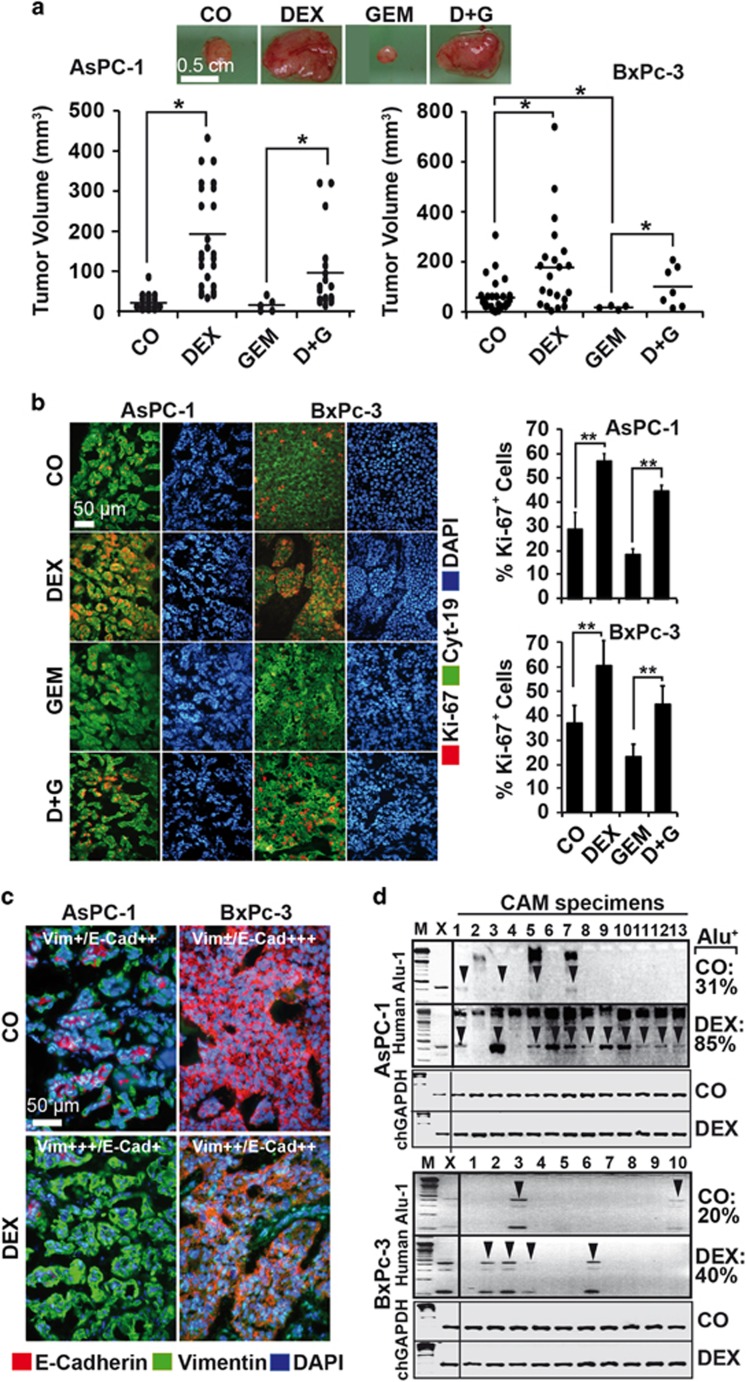
Dexamethasone induces progression and metastasis of egg tumor xenografts. (**a**) The established pancreatic cancer cell lines AsPC-1 and BxPc-3 cells were left untreated (CO) or were precultured with dexamethasone (DEX, 1 *μ*M) for 48 h. Then, 50 nM gemcitabine was added to dexamethasone pretreated or untreated cells (D&G or GEM, respectively) for 72 h, followed by transplantation of 5 × 10^5^ viable cells to the CAM of fertilized chicken eggs at day 9 of embryonic development. After 9 days, the tumors were resected, and the volumes determined and presented as single dots and the means per group in the diagram. Representative images of BxPc-3 xenografts are shown above the diagrams, *P<0.05. (**b**) Double immunofluorescence staining of AsPC-1 and BxPc-3 xenograft tissue with human-specific antibodies against the cytoskeletal protein cytokeratin 19 (Cyt-19, green) and the proliferation marker Ki-67 (red). The cell nuclei were counterstained with DAPI (blue). The cell sections were analyzed under × 100 magnification, and representative images are shown. The number of Ki-67-positive cells was quantified in 10 visual fields at × 400 magnification, and the means±S.D. are shown, **P<0.01. (**c**) Double immunofluorescence staining of xenograft tissue with human-specific antibodies against E-cadherin (red) and vimentin.^29^ The cell nuclei were counterstained with DAPI (blue). The cell sections were analyzed under × 400 magnification, and representative images are shown. For evaluation of the expression levels, a semiquantitative scoring system was used, as described in Figure 1d. (**d**) Genomic DNA was isolated from freshly resected CAM tissues (AsPC-1 *n*=13, BxPc-3 *n*=10), followed by PCR analysis with primers for human Alu sequences. Positive bands (arrows) were identified by their size of 4000 basepairs that was identified by comparison with a DNA marker (M). DNA isolated from xenograft tumors (X) served as positive control. The percentage of Alu-positive bands before (CO) and after dexamethasone treatment (DEX) is shown on the right. Internal chick GAPDH controls (500 basepairs) for the Alu-PCRs are shown below the human Alu sequences

To confirm these results and to examine the effect of dexamethasone to standard chemotherapy, we treated AsPC-1, BxPc-3 and MIA-PaCa2 cells with dexamethasone, gemcitabine or both together, or let the cells untreated, before they were transplanted to the CAM of fertilized chicken eggs. After 9 days, large tumor xenografts developed in all dexamethasone-treated groups, even in the presence of gemcitabine, whereas the tumor sizes in groups that have not received dexamethasone were smaller and almost undetectable after gemcitabine treatment (Figure 2a and Supplementary Figure S1a). Dexamethasone usage was associated with enhanced proliferation and the combination with gemcitabine prevented it, as found by Ki-67 immunofluorescence staining and counting of positive cells (Figure 2b and Supplementary Figure S1b). In addition, the expression of E-cadherin was inhibited and the expression of vimentin was increased upon dexamethasone treatment (Figure 2c and Supplementary Figure S1c), together with an enhanced invasive potential, as concluded from PCR detection of human Alu sequences in genomic DNA from chick CAM and liver. Whereas 31% of the CAM and 0% of the liver tissues were Alu positive in eggs with untreated AsPC-1 cells, dexamethasone increased the percentage to 85% and 60%, respectively (Figure 2d and Supplementary Figure S1d). Similarly, dexamethasone increased the invasion of BxPc-3 and MIA-PaCa2 tumor cells to the CAM from 20% to 40% and from 30% to 70%, respectively (Figure 2d and Supplementary Figure S1e). Internal chicken GAPDH controls were performed and the results confirmed equal conditions (Supplementary Figure S1f). No significant differences in the mean weights of the embryos occurred between the groups (Supplementary Figure S1g), suggesting that no toxic side effects occurred.

To further highlight the influence of dexamethasone to tumor progression, the cells were treated with dexamethasone, followed by western blot analysis. Each cell line exhibited an already basal expression of the glucocorticoid receptor and dexamethasone further induced (MIA-PaCa2, BxPc-3) or repressed (AsPC-1) it (Supplementary Figure S2a). This implies a functional autoregulation of the glucocorticoid receptor by its ligand, as previously described.^30^ Dexamethasone switched the epithelial cell morphology to a more spindle-shaped phenotype, as shown by microscopy 240 h after dexamethasone treatment (Supplementary Figure S2b). The migratory activity increased significantly upon dexamethasone treatment, as measured by scratch and transwell assays (Supplementary Figures S2c and d). This was accompanied by the inhibition of E-cadherin and the induction of vimentin in AsPC-1 and BxPc-3 cells, as detected by double immunofluorescence staining (Supplementary Figure S2e). Dexamethasone did not alter the expression of E-cadherin and vimentin in MIA-PaCa2 cells because of already high basal expression levels. These results suggest that dexamethasone induces EMT-related tumor progression.

### Dexamethasone induces TGF*β* signaling

**Figure 3:**
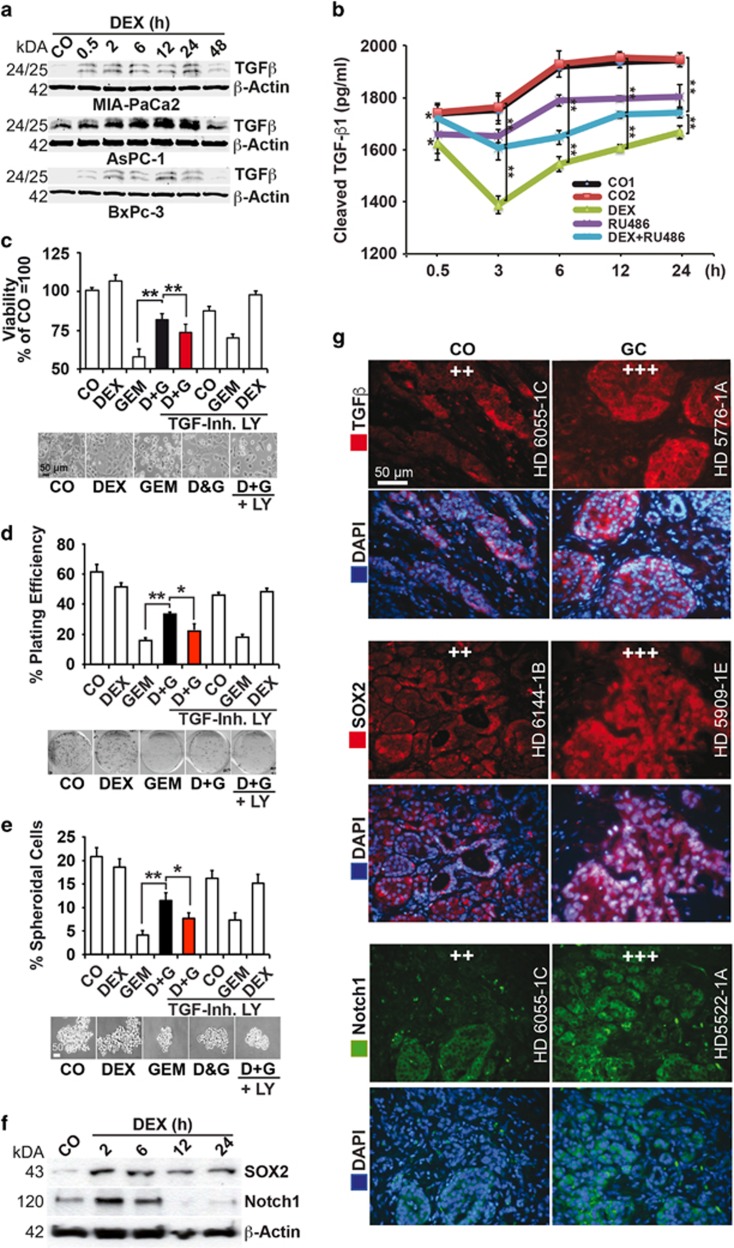
Dexamethasone induces TGF*β*-associated EMT and metastasis ***in vitro***. (**a**) The cells were treated with 1 *μ*M dexamethasone (DEX) for 0.5 to 48 h as indicated, and the expression of TGF*β* was detected by western blot analysis as described above. (**b**) The amount of soluble TGF*β*1 ligand (pg/ml) in AsPC-1 cell culture supernatant was examined by ELISA assay after dexamethasone (DEX, 1 *μ*M) treatment as described above. The glucocorticoid receptor antagonist mifepristone-RU486 (RU, 1 *μ*M) was added 30 min before dexamethasone treatment. Vehicle controls were CO1 (EtOH 1 : 25 000 for DEX) and CO2 (EtOH 1 : 12 500 for DEX and RU486). Error bars are shown together with the significance between DEX and CO1 and between DEX+RU486 and CO2. *P<0.05 and **P<0.01. The values were calibrated to the NIBSC/WHO TGF-*β*1 International Reference Reagent 87/514. (**c**) AsPC-1 cells were left untreated (CO) or were precultured in 1 *μ*M dexamethasone (DEX) for 48 h. Then, 50 nM gemcitabine was added to untreated cells (GEM), and DEX-treated cells (D&G) for another 48 h in the presence or absence of the TGF*β*R-I/II kinase inhibitor LY2109761 (TGF-Inh. LY, 10 *μ*M) that was administered together with dexamethasone to the groups indicated. The viability was measured by MTT assay. The percentage of viable cells in the control groups was set to 100%, **P<0.01. Representative photographs of morphology are shown below the diagrams in the mentioned five groups. (**d**) ASPC-1 cells were treated as described above. At 48 h after gemcitabine treatment, 400 viable cells/well were seeded at clonal density into 6-well plates. The cells were grown without a change of medium for 2 weeks, followed by the evaluation of fixed and Coomassie-stained colonies consisting of 50 cells at least. The plating efficiency, expressed as a percentage, was calculated by the following formula: 100 × number of colonies/number of seeded cells, *P<0.05, **P<0.01. Representative photographs of colonies 2 weeks after the incubation are shown below the diagrams in the mentioned five groups. (**e**) AsPC-1 cells were treated as described above and then seeded in ultra-low attachment plates at a low density of 1 × 10^4^ cells/ml in serum-free, growth factor-containing medium for spheroid formation. After 10 days, photographs were obtained and photographed under × 100 magnification. To detect the number of spheroidal-growing cells, the spheroids were dissolved, and the single cells were counted. The percentage of spheroidal-growing cells relative to the seeded cells was evaluated, *P<0.05, **P<0.01. Representative photographs of spheroids are shown below the diagrams in the mentioned five groups. (**f**) Cells were left untreated (CO) or were cultured in 1 *μ*M dexamethasone (DEX) for 2 to 24 h as indicated, followed by examination of SOX2 and Notch1 expression by western blot analysis. (**g**) Representative paraffin-embedded sections (HD 6065-1C, HD 5776-1A, HD 6144-1B, HD 5909-1E, HD 6055-1C, HD 5522-1A) derived from primary PDA tissue from patients with documented preoperative administration of glucocorticoids (GC, *n*=17) or not (CO, *n*=17) were evaluated by immunofluorescence staining to detect the expression of TGF*β* (red), SOX2 (red) and Notch1.^29^ The cell nuclei were counterstained with DAPI (blue). The sections were analyzed under × 400 magnification, and representative images are shown. For evaluation of the expression levels, a semiquantitative scoring system was used, as described in Figure 1d

Because TGF*β* is a key player in EMT, we measured its expression by western blot analysis.^31^ Dexamethasone induced the expression of the 25/26 kDa form of mature TGF*β* protein in AsPC-1, MIA-PaCa2 and BxPc-3 cells within 0.5 h and further induced it during the subsequent 24 h, followed by downregulation at 48 h (Figure 3a). This was associated with a decrease of soluble TGF*β*1 protein in the cell supernatant after dexamethasone that may be explained by receptor binding and as detected by ELISA assay (Figure 3b and Supplementary Figure S3a). In contrast, coincubation of the glucocorticoid receptor antagonist mifepristone (RU486) counteracted the dexamethasone-induced dropdown of soluble TGF*β*1. These results are underlined by coincubation experiments with the TGF*β* receptor-I and -II inhibitor LY2109761 that significantly revised the dexamethasone-induced gemcitabine resistance, as measured in AsPC-1 cells by assays for viability, clonogenicity and spheroid formation (Figures 3c–e and Supplementary Figures S3b–d). Remarkably, dexamethasone did not induce gemcitabine resistance upon cotreatment of cells shortly after detachment of the cells from the culture dish with trypsin (data not shown), suggesting that trypsin removed membrane-associated dexamethasone-binding partners, for example, membrane-bound glucocorticoid receptor and other cell surface receptors. To obtain knowledge whether cancer progression was induced, we treated the cells 48 h after trypsinization with dexamethasone for 2, 6, 12 and 24 h, followed by western blot analysis of the pluripotency markers SOX2 and Notch1 and saw that dexamethasone rapidly induced the expression of both proteins (Figure 3f and Supplementary Figure S3e). To verify these data in tissues from patients, we performed immunofluorescence staining. Fluorescence microscopy revealed a higher expression of TGF*β*, SOX2 and Notch1 in tissue of patients who received a glucocorticoid before surgery (*n*=17) compared with tissue of patients who received no glucocorticoid ((*n*=17, Figure 3g and Table 1). Because diverse glucocorticoids were administered to patients, these results suggest that not only dexamethasone but also other glucocorticoids induce TGF*β*1-dependent EMT and tumor progression.

### Dexamethasone mediates Ras/JNK and AP-1 signaling

**Figure 4:**
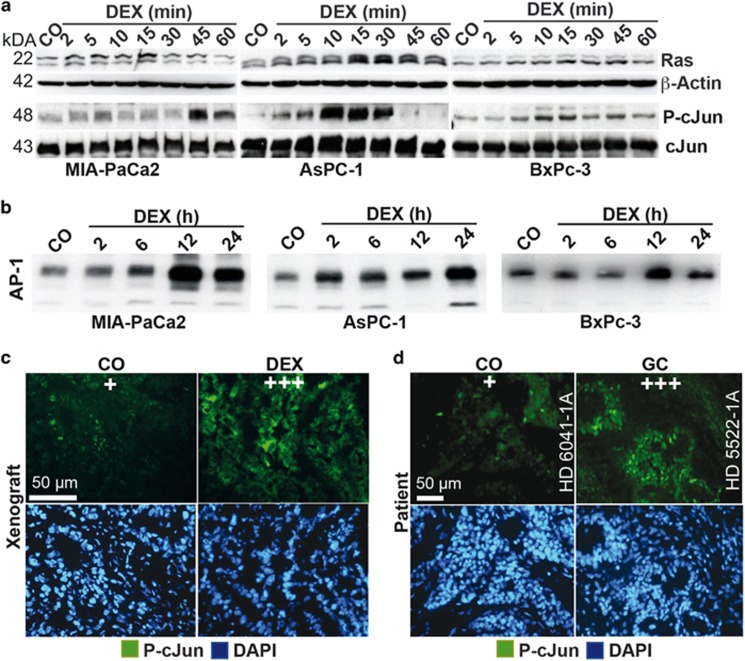
Dexamethasone induces rapid Ras/JNK and cJun/AP-1 signaling. (**a**) MIA-PaCa2, AsPC-1 and BxPc-3 cells were left untreated (CO) or were cultured in 1 *μ*M dexamethasone (DEX) for 2 to 60 min as indicated. Then, cells were quickly put on ice and protein extracts were prepared. The expression of Ras was detected by western blot analysis. *β*-Actin served as loading control. The phosphorylation of cJun at Serin 63 (P-cJun) was examined by a JNK/SAP kinase assay and phosphorylated cJun (P-cJun was detected by SAPK/JNK kinase assay. Nonphosphorylated cJun served as a control for equal conditions). (**b**) The cells were left untreated (CO) or were treated with 1 *μ*m dexamethasone (DEX), and at the time points indicated nuclear protein extracts were harvested. The DNA binding was analyzed by EMSA, using a specific biotin-labeled oligonucleotide probe of the AP-1 consensus sequence as present in the vimentin promoter. (**c**) Tissue sections from human primary PDA xenografts growing on mice, as described in Figures 1a and b were stained with an antibody specific for phosphorylated cJun to detect the expression of P-cJun^29^ by immunofluorescence microscopy at × 400 magnification, and representative images are shown. The cell nuclei were counterstained with DAPI (blue). (**d**) Representative paraffin-embedded sections (HD 6041-1A, HD 5522-1A) derived from primary PDA tumor tissue from patients with documented preoperative administration of glucocorticoids (GC, *n*=17) or not (CO, *n*=17) were evaluated by immunofluorescence for expression of P-cJun.^29^ The cell nuclei were counterstained with DAPI (blue). The sections were analyzed under × 400 magnification, and representative images are shown. For evaluation of the expression levels, a semiquantitative scoring system was used, as described in Figure 1d

To measure rapid membrane and kinase effects of dexamethasone, we detected the expression of Ras/JNK signaling and AP-1 activity, known to activate TGF*β* expression.^32^ Dexamethasone led to enhanced RAS expression and phosphorylation of cJun within 2 to 5 min in MIA-PaCa2, AsPC-1 and BxPc-3 cells, with peaks at 10 to 45 min, as measured by western blot analysis and JNK/SAP kinase assays (Figure 4a). An enhanced binding activity of the transcription factor and JNK-target cJun/AP-1 occurred within 2 h, with peaks at 12 and 24 h as detected by EMSA and oligonucleotides of the vimentin promoter AP-1 binding site (Figure 4b). Immunofluorescence staining of human PDA tissue transplanted to mice revealed that the expression of P-cJun was still increased at late time points after dexamethasone treatment (Figure 4c, compare Figures 1a and b). Similar results were obtained by staining of patient tissue that demonstrated a higher expression of P-cJun upon intake of a glucocorticoid before surgery (Figure 4d and Table 1).

**Figure 5:**
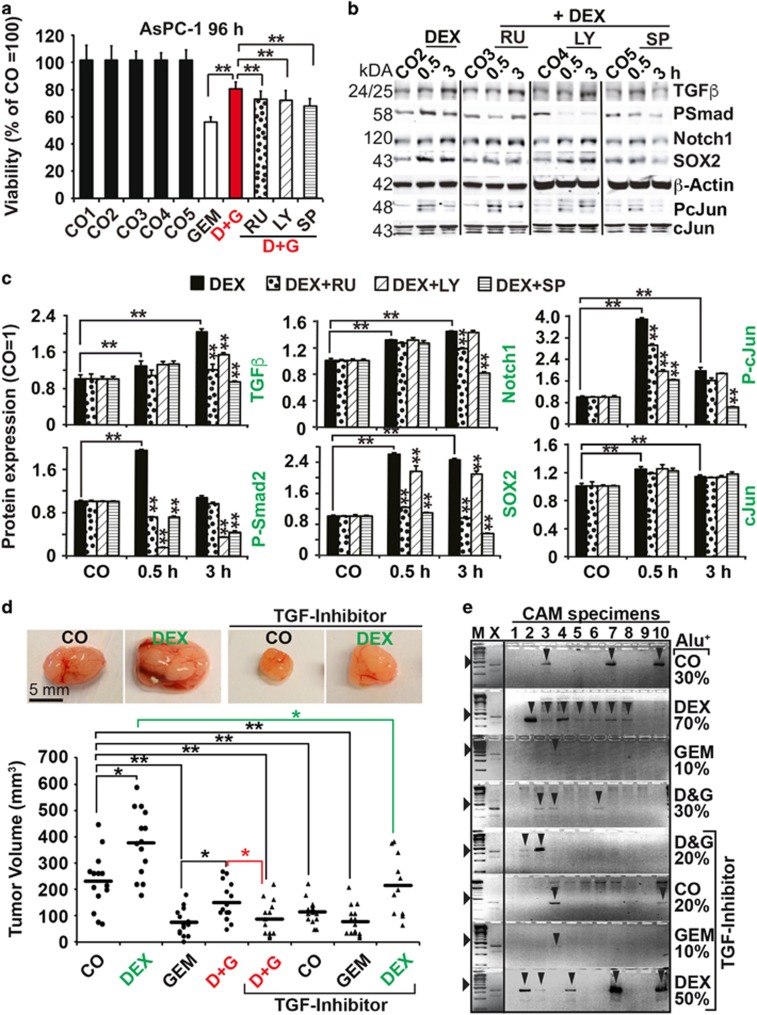
Dexamethasone mediates tumor progression by a complex signaling network. (**a**) AsPC-1 cells were left untreated (CO1) or were treated with vehicle alone (CO2: EtOH 1 : 25 000; CO3 EtOH 1 : 12 500; CO4 EtOH 1 : 12 500+DMSO 1 : 1000; CO5 EtOH 1 : 12 500+DMSO 1 : 2000) or with mifepristone/RU486 (RU, 1 *μ*M), LY2109761 (LY, 10 *μ*M) and SP600125 (SP, 10 *μ*M). After 30 min, 1 *μ*M dexamethasone (DEX) was added. After 48 h, 50 nM gemcitabine was added to the respective groups (GEM, D&G) for additional 96 h, followed by MTT assay. The percentage of viable cells in the control groups was set to 100%, and the significance was evaluated. **P<0.01. (**b**) AsPC-1 cells were left untreated or were treated with mifepristone/RU486 (RU, 1 *μ*M), LY2109761 (LY, 10 *μ*M) and SP600125 (SP, 10 *μ*M) and the respective vehicle controls. After 30 min, 1 *μ*m dexamethasone (DEX) was added to the groups indicated and the expression of TGF*β*, P-Smad2, Notch1, SOX2, P-cJun and cJun was examined by western blot analysis. *β*-Actin served as loading control. (**c**) Column diagrams depicting the relative protein levels of the western blot bands above, as detected by Image Studio (Li-COR Biosciences GmbH, Bad Homburg vor der Höhe, Germany) after normalization to *β*-actin. The pixel density in the controls was set to 1, *P<0.05, **P<0.01. (**d**) MIA-PaCa2 cells were treated as described in Figure 3c, followed by transplantation of 1 × 10^6^ viable cells to fertilized chicken eggs (*n*=15 per group) at day 9 of embryonic development. The tumor xenografts were analyzed as described in Figure 2a. Representative images of the tumor sizes are shown, *P<0.05, **P<0.01. (**e**) Genomic DNA was isolated from CAM (*n*=10) tissue directly adjacent to the tumor xenografts and the expression of human Alu sequences was evaluated as described in Figure 2d

To evaluate the impact of each key player, we treated AsPC-1 cells with dexamethasone and gemcitabine alone or together and in the presence or absence of inhibitors of the glucocorticoid receptor (mifepristone/RU486), the TGF*β* receptor (LY2109761) or JNK signaling (SP600125). Each inhibitor alone partially reversed the dexamethasone-mediated gemcitabine resistance as examined 72 and 96 h after treatment by MTT assay (Figure 5a and Supplementary Figure S4a). Western blot analysis shows that RU486 inhibited the dexamethasone-induced upregulation of TGF*β* and its mediator P-Smad2, along with inhibition of Notch1, SOX2 and P-cJun expression (Figures 5b and c). LY2109761 inhibited the upregulation of TGF*β*, P-Smad2, SOX2 and P-cJun, but had no effect on Notch1 expression. Similarly, SP600125 inhibited the activation of all examined proteins. These results were verified *in vivo* in MIA-PaCa2 cells and by the use of the TGF*β* receptor inhibitor (Figure 5d). AsPC-1 cells were treated with dexamethasone and gemcitabine alone or together, ±LY2109761, followed by xenotransplantation of equal amounts of viable cells to the CAM of fertilized chicken eggs. At 10 days after transplantation, the tumors were resected and the tumor volumes were detected by calipers. In confirmation with our *in vitro* results, the inhibition of TGF*β* signaling reversed the dexamethasone-induced gemcitabine resistance. The measurement of the invasion potential by Alu-PCR showed that 70% of CAM tissues from dexamethasone groups were Alu positive, but LY210976 reduced the percentage to 50% (Figure 5e). Accordingly, 30% of CAM tissues from the dexamethasone and gemcitabine combination groups were Alu positive, but LY210976 reduced it to 20%. Internal chicken GAPDH controls for Alu PCR reactions demonstrated equal conditions (Supplementary Figure S4b). These results suggest that a dexamethasone-induced complex signaling network mediates tumor progression with TGF*β*, RAS/JNK and AP-1 as key players.

## Discussion

**Figure 6:**
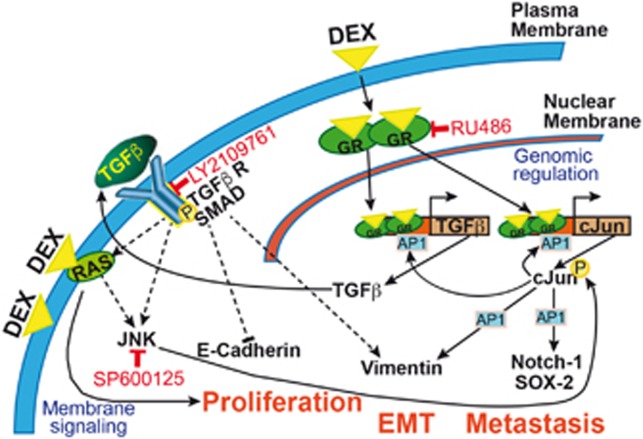
Model of dexamethasone mediated cross-signaling leading to tumor progression. Dexamethasone (DEX) enters the cytoplasm and binds to the glucocorticoid receptor (GR) to form glucocorticoid–glucocorticoid receptor homodimers that enter the nucleus, interact with other transcription factors such as cJun/AP-1 and bind to glucocorticoid receptor element-containing target genes, involving TGF*β* and cJun. This activates the expression of TGF*β* and cJun/AP-1. cJun/AP-1 binds to its own and to the TGF*β* promoter and reinforces the expression. Besides, cJun/AP-1 binds to other target genes and thereby activates, for example, the expression of Notch-1, SOX-2 and vimentin. Soluble TGF*β* ligand is secreted and binds to the surface receptor TGF*β*R-I/II kinase complex, leading to the intracellular recruitment and phosphorylation of Smad proteins. Activated Smad proteins associate with Smad4 and translocate to the nucleus, where they interact with transcription factors, coactivators, corepressors and chromatin remodeling factors that control the expression of numerous target genes. As a consequence, E-cadherin and vimentin are expressed. Soluble TGF*β* ligand can also activate other surface membrane receptors and thereby activates Ras/JNK signaling that contributes to the scenario by increasing cJun/AP-1 expression. All these amplifying mechanisms together may converge into proliferation, EMT and metastasis. The glucocorticoid receptor antagonist mifepristone (RU486), the TGF*β*R-I/II kinase inhibitor LY2109761 and the JNK inhibitor SP600125 interfere with this crosstalk, but none of the inhibitors on their own are able to completely block it

Whereas glucocorticoids have many advantages for cancer patients, our present results hint to undesirable side effects in PDA, namely the induction of tumor progression, chemotherapy resistance and metastasis by a complex sequence of signaling (Figure 6).

We assume that dexamethasone-induced therapy resistance and tumor progression depends on interactions with membrane-associated factors, for example, the membrane-bound glucocorticoid receptor coupled to G proteins, and the activation of downstream signaling.^33^ This assumption is based on our observation that freshly trypsinized cells did not respond with tumor progression to dexamethasone treatment—most likely because trypsin removed membrane-associated binding partners from the cell surface.^34^ Such membrane-mediated actions of glucocorticoids were already observed in lower vertebrate species and are believed to represent the more evolutionarily ancient forms of glucocorticoid receptor signaling.^33^ This very old way might have become dominant in PDA cells during the dedifferentiation process of normal cancer cells into CSCs. Several recent reports support our observation of dexamethasone-induced EMT and cancer progression. For example, elevated JNK activity in breast cancer did not induce apoptosis as expected, but cell migration and EMT, along with an enhanced expression of vimentin and AP-1.^35^ In addition, targeting of RAS/JNK eliminated CSC features and prevented tumor formation in pancreatic cancer.^36^ Similarly, a pivotal role of JNK in the maintenance of self-renewal and tumorigenicity in glioma stem-like cells was demonstrated.^37^ Moreover, AP-1 was defined as a critical regulator of a complex program of gene expression mediating an invasive phenotype.^27^

However, there are reports that published the opposite effects. One study for example claimed that dexamethasone inhibited the invasiveness of a human pancreatic cancer cell line that was examined by *in vitro* invasion assay and expression of MMP-9.^38^ Another study found a dexamethasone-induced inhibition of *in vitro* invasion of the PDA cell line Panc-TuI and, after orthotopic transplantation to immunodeficient mice, reduced tumor growth and metastasis.^39^ We do not know the reason for these obvious discrepancies to our results, but suspect that it might be because of differences in cell lines, experimental conditions and data evaluation. For example, the conclusions are drawn from one cell line only in both studies. In addition, the *in vitro* invasion assays are highly susceptible to faults, the presented pictures are out of focus and not quantitatively evaluated. Next, the PDA cell line Panc-TuI was used that we never evaluated, and therefore it might well be that this is one of a few solid tumor cells that are sensitive to dexamethasone treatment. In the orthotopic xenograft experiments, dexamethasone was not applied to inhibit primary tumor growth as in our study, but first when the xenografts were resected with the intention to prevent a relapse.^39^ Moreover, orthotopic transplantation to the mouse pancreas by median laparatomy is known to result in xenograft tumors that strongly vary in size. Thus, the measurement of an increase in tumor volume over time by *in vivo* imaging and the presentation of the results as ‘mean increase of tumor volume over time’ may provide the most accurate results. However, Egberts *et al.*^39^ evaluated tumor growth only once at the end of the experiment and presented the results as ‘mm^3^’. An explanation of how the tumor volumes were evaluated was not provided and hence we are not able to judge the method.

Signaling by the cytoplasmic glucocorticoid receptor was involved in our system as well, and may have mediated the enhanced expression of TGF*β* by binding to the glucocorticoid-responsive element in the TGF*β* promoter,^19^ as concluded from partial inhibition by mifepristone/RU486. Similarly, glucocorticoid receptor/AP-1 interactions might have enforced the signaling cascade by the binding of AP-1 to respective binding sites in the TGF*β* and vimentin promoters,^40^ and to other target genes that are involved in stemness and progression. Positive feedback loops mediated by autoactivation of cJun transcription by AP-1 and the subsequent activation of TGF*β*, which in turn activates Ras/JNK signaling and cJun activation by phosphorylation, have enforced the scenario.^25^ Our results obtained after inhibition of JNK signaling confirmed a direct link of dexamethasone-induced rapid and most likely nongenomic TGF*β* and RAS/JNK action,^41^ with subsequent cJun/AP-1-mediated upregulation of vimentin,^42^ along with downregulation of E-cadherin.^43^ Interestingly, we observed that the amount of soluble TGF*β*1 ligand in cell culture supernatant of dexamethasone treatment was diminished, hinting to the binding of TGF*β*1 to its receptor. This assumption is underlined by our result that a TGF*β* receptor I/II blocker partially neutralized the dexamethasone-induced tumor progression. We do not exclude that a complex network of other signaling cascades is involved, such as glucocorticoid receptor-chromatin interactions, that are described to require AP-1 as a major partner.^44^ In this regard, the loss of glucocorticoid receptor in transgenic mice resulted in tumorigenesis because of defective chromosome segregation.^18^

We observed the induction of RAS/JNK signaling by dexamethasone, whose relation to dexamethasone-induced anti-apoptotic signaling in breast cancer by interaction with protein kinases PI3-K and Akt is well known.^45^ This matches to our data, because PI3-K and Akt are the best-characterized RAS effectors.^26^ Other involved key players may be the serum and glucocorticoid-regulated kinase-1 (SGK1), as reported by Conzen and colleagues^10, 46, 47, 48^ in breast, ovarian and prostate cancer. In a human breast cancer xenograft model, the inhibition of glucocorticoid receptor signaling reversed the glucocorticoid-induced chemotherapy resistance,^10^ in line with our results, where the inhibition of the glucocorticoid receptor partially reversed the dexamethasone-induced gemcitabine resistance.

In summary, we suggest a careful weighing of advantages and disadvantages of glucocorticoids in therapy of solid tumors and the use of alternatives such as nonsteroidal medicaments for treatment of inflammation, emesis, edema, allergies and immune response. Our data may be of clinical relevance with regard to the still frequent use of glucocorticoid medication, but also considering the known elevated levels of endogenous glucocorticoids in chronic pancreatitis, under severe stress conditions, in chronic depression, after protein-rich meals and acute smoking.^49^

## Materials and methods

### Cell culture

The human established PDA cell lines MIA-PaCa2, AsPC-1 and BxPc-3 were obtained from the American Type Culture Collection (Manassas, VA, USA) and were cultured as previously described.^50^

### Reagents

Stock solutions of dexamethasone (25 mM, ≥98% pure, Sigma-Aldrich Chemie, Munich, Germany) and mifepristone (RU486, 50 mM, ≥98% pure, Sigma-Aldrich) were prepared in ethanol. LY2109761 (10 mM, ≥99% pure, Selleckchem, Munich, Germany) and SP600125 (20 mM, ≥99% pure, Selleckchem) were prepared in DMSO. Gemcitabine (126 mM, Lilly Deutschland, Bad Homburg, Germany) was freshly diluted in cell culture medium to prepare a 100 *μ*M stock solution. The final concentrations of the solvents in media were 0.1% or less.

### Cell viability

Cell viability was measured using 3-(4,5-dimethylthiazol-2-yl)-2,5-diphenyltetrazolium bromide (MTT) as previously described.^50^

### FACS analysis

The cells were treated with FITC-conjugated annexin-V (Life Technologies, Darmstadt, Germany), and FACS analysis was performed as previously described.^50^

### Colony assay

At 48 h after treatment, the cells were reseeded in complete medium in 6-well tissue culture plates. The colonies were evaluated 14 days later, as previously described.^50^

### Spheroid assay

Cells were cultured in human NeuroCult NS-A serum-free medium with supplements (STEMCELL Technologies, Cologne, Germany), and analysis of spheroid formation was performed as previously described.^50^

### Western blot

Western blot was performed as previously described.^50^ The antibodies were: mouse monoclonal against vimentin (V9, Abcam, Cambridge, UK), phosphorylated (P)-p44/42 MAPK (Cell Signaling Technology, Cambridge, UK), *β*-actin (Sigma-Aldrich, Munich, Germany), rabbit monoclonal against E-cadherin (24E10, Abcam), Ras (EPR3255, Abcam), Notch1 (Cell Signaling), rabbit polyclonal against TGF*β*1/2/3 (Cell Signaling), SOX2 (Abcam), phosphorylated Smad2 (Life Technologies, Darmstadt, Germany), cJun and phosphorylated cJun (P-cJun) (Cell Signaling), glucocorticoid receptor (H-300, Santa Cruz Biotechnology, Heidelberg, Germany) and secondary goat anti-mouse or goat-anti rabbit IgG horseradish peroxidase-conjugated antibodies (Santa Cruz).

### TGF*β*1 ELISA

TGF-*β*1 concentrations in supernatant were determined according to the ELISA kit instructions of the manufacturer (Enzo Life Sciences, Lörrach, Germany).

### SAP/JNK assay

JNK/SAP kinases were immunoprecipitated from cell extract, and a kinase reaction was performed according to the instructions of the manufacturer (New England Biolabs, Frankfurt, Germany). Phosphorylated cJun was detected by western blot using a rabbit polyclonal antibody specific for cJun phosphorylated at serine 63. Mouse mAb cJun (L70B11, New England Biolabs) served as a control for equal conditions.

### Immunohistochemistry

Staining was performed on established cell lines growing in chambers of the Nunc Lab-Tek Chamber Slide system (Sigma-Aldrich Chemie), or on 6 *μ*m frozen or paraffin-embedded tissue sections as previously described.^50^ The antibodies were mouse monoclonal against cytokeratin (Cyt)-19 (Abcam), rabbit monoclonal against E-cadherin (Cell Signaling Technology), Ki-67 (Abcam), Notch1, P-cJun (Cell Signaling), goat polyclonal against vimentin (R&D Systems, Wiesbaden-Nordenstadt, Germany), SOX2 (Santa Cruz), c-Met (Biozol, Eching, Germany) and TGF*β*1 (Acris, Herford, Germany).

### Scratch assay

Cells (6 × 10^5^) were seeded in 6-well plates and grown to confluence overnight. A line was scraped within the cell layer using the fine end of a 10 *μ*l white pipette tip. Images of migrating cells were obtained with a Nikon Eclipse TS100 microscope (Nikon, Tokyo, Japan).

### Transwell assay

Cells (10^5^ cells/cm^2^) were seeded in 24-well plates with transwell polycarbonate filters (8 mm, Corning Life Sciences, Amsterdam, The Netherlands). The number of transmigrated cells was counted under a Nikon Eclipse TS100 microscope.

### EMSA

Nuclear protein extracts were prepared with the NucBuster Protein Extraction Kit (Merck, Darmstadt, Germany), and the bandshift reaction was performed using the Light Shift Chemiluminescent EMSA Kit, according to the manufacturer’s instructions (Thermo Fisher Scientific, Bonn, Germany). Biotin 3' end-labeled oligonucleotides or unlabeled oligonucleotides (MWG Biotech, Ebersberg, Germany), corresponding to the vimentin AP-1 site at the promoter region -715/-676,^51^ were: AP-1-sense: 5'- AGGGCGCGGTGAGTCACCGCCGGTGACTAAGCGACCCCAC-3' and AP-1-antisense: 5'-GTGGGGTCGCTTAGTCACCGGCGGTGACTCACCGCGCCCT-3' (Consensus AP-1 sites are underlined).

### Xenotransplantation to chicken eggs

Tumor cells were transplanted to the chorioallantoic membrane^52^ of fertilized eggs from genetically identical hybrid LB chicken, followed by treatment and evaluation of tumor take and volume as previously described.^50^

### Alu PCR

Genomic DNA was isolated from chick tissue using the DNeasy Blood and Tissue Kit (Qiagen, Hilden, Germany), and PCR was performed with primers for human Alu sequences: Alu-sense: 5'-GTAAGAGTTCCGTAACAGGACAGC T-3' and Alu-antisense: 5'-CCCCACCCTAGGAGAACTTCTCTTT-3' (MWG Biotech, Ebersberg, Germany). Chicken GAPDH-sense: 5'-GAGGAAAGGTCGCCTGGTGGATCG-3' chicken GAPDH-antisense: 5'-GGTGAGGACAGGCAGTGAGGAACG-3' (MWG Biotech).

### Tumor xenotransplantation to immunodeficient mice

The primary PDA xenograft line T30 was isolated from surgical specimens, which were mechanically minced, and transplanted into the flanks of NMRI (nu/nu) mice, followed by subtransplantation as recently described.^53^ Cancer spheres were isolated from T30 xenografts and cultured in NeuroCult NS-A serum-free medium with supplements (STEMCELL Technologies, Cologne, Germany), followed by *in vitro* treatment and subcutaneous injection into the left and right flanks of 6-week-old NMRI (nu/nu) male mice, 6 animals per group. The tumor volumes (*V*) were determined 3 weeks after transplantation using two diameters and were calculated with the formula: *V*=1/2 (length × width^2^). The experiments were performed in the animal facilities of the University of Heidelberg after receiving approval from the authorities (Regierungspräsidium Karlsruhe, Karlsruhe, Germany).

### Statistical analysis

All *in vitro* experiments except the colony-forming assays were performed three times in triplicate (*n*=9), and the quantitative data are presented as the means±S.D. The colony-forming assays were performed twice in sextuplicate (*n*=12). For the *in vivo* transplantation experiments, 20 eggs (*n*=20) or 6 mice with tumors on both flanks per group (*n*=12) were used. The significance of the data was analyzed with Student’s *t*-test. Variances in the tumor volumes were evaluated with the Kruskal–Wallis test and the Mann–Whitney test with Bonferroni correction. *P<0.05 was considered statistically significant and **P<0.01 was considered highly statistically significant.

## Publisher’s Note

Springer Nature remains neutral with regard to jurisdictional claims in published maps and institutional affiliations.

## Figures and Tables

**Table 1:** Protein expression in patient PDA tissues

	**Patient ID**	**Viment**	**E-Cad**	**SOX2**	**Notch1**	**TGF*****β***	**P-cJun**
*Controls without glucocorticoid treatment*	HD 5921-1B	++	+++	++	++	++	++
	HD 6040-1A	++	++	+++	+	++	++
	HD 6041-1A	++	+++	++	+	++	++
	HD 6055-1C	+++	+++	++	++	+	+
	HD 6065-1A	+	+++	++	++	++	+
	HD 6122-1A	++	+++	++	++	+	+
	HD 6137-1A	+	++	+++	+	+	+
	HD 6144-1B	++	+++	+	+	++	+
	HD 6121-1A	++	+++	+	++	++	+
	HD 6127-1A	++	+++	++	+	+	++
	HD 5913-1B	++	+++	++	++	++	++
	HD 6053-1B	++	+++	++	+	+	++
	HD 6069-1A	+++	+++	++	+	+	+
	HD 6034-1A	++	++	++	++	++	+
	HD 6026-1A	+	+++	+	+	++	+
	HD 5897-1A	+	+++	+	+	+	+
	HD 5934-1B	+	+++	++	+	+	+
	Average	++	+++	++	+	++	+
*Glucocorticoid treatment*							
Fluticason-17 propionate 250 *μ*g	HD 5174-1A	+++	+	++	+++	+	++
Fluticasone furoate 100 *μ*g	HD 5266-1A	+++	+	+++	++	+++	+++
Budesonide 3 mg	HD 5353-1A	+++	++	++	++	++	+++
Prednisolone 2 mg	HD 5522-1A	+++	+	+++	+++	++	++
Beclometasone dipropionate 100 *μ*g	HD 5676-1A	++	+	+++	+++	+++	+++
Prednisolone 5 mg Prednisolone 1 mg	HD 5746-1A	+++	++	+++	+++	+++	+++
Budesonide 200 *μ*g	HD 5909-1E	+++	++	++	++	++	+
Prednisone 20 mg	HD 6133-1A	+++	++	+++	+++	++	++
Beclometasone dipropionate 100 *μ*g Fluticasone furoate 27.5 *μ*g	HD 5082-2A	+++	++	++	+++	++	++
Budesonide 400 *μ*g	HD 5954-1A	+++	+	+++	++	+++	+++
Prednisone 5 mg	HD 5025-1A	+++	++	+++	+++	+++	+++
Budesonide 200 *μ*g	HD 5777-1A	+++	++	++	+++	+++	++
Budesonide 100 *μ*g	HD 5833-2A	+++	+	+	+++	++	+
Budesonide 200 *μ*g	HD 5853-1B	+++	+++	+++	+++	+++	+++
Prednisone 5 mg	HD 5936-1A	+++	++	+++	+++	+++	+++
Budesonide 200 *μ*g	HD 5858-1A	+++	++	+++	++	++	+++
Budesonide 200 *μ*g	HD 5808-1A	++	+	++	++	+++	+++
	Average	+++	++	+++	+++	+++	+++

Prednisolone, beclometasone dipropionate/fluticasone furoate and budesonide were taken either orally or were inhaled. Marker expression was determined by immunohistochemistry and fluorescence microscopy. The intensity of fluorescence and the percentage of positive cells were determined by double-blind visual judgment: very strong expression and very high percentage (≥75%) (++++), strong expression and high percentage (≥50%) (+++), moderate expression and medium percentage (≥25%) (++), weak expression and low percentage (≤25%) (+) and absent (−).
