# Supplementary figures and images for: Dexamethasone mediates pancreatic cancer progression by glucocorticoid receptor, TGFβ and JNK/AP-1
Source: Cell Death Dis. 2017 Oct 5;8(10):e3064–. doi: 10.1038/cddis.2017.455 (PMC5680577; doi:10.1038/cddis.2017.455)

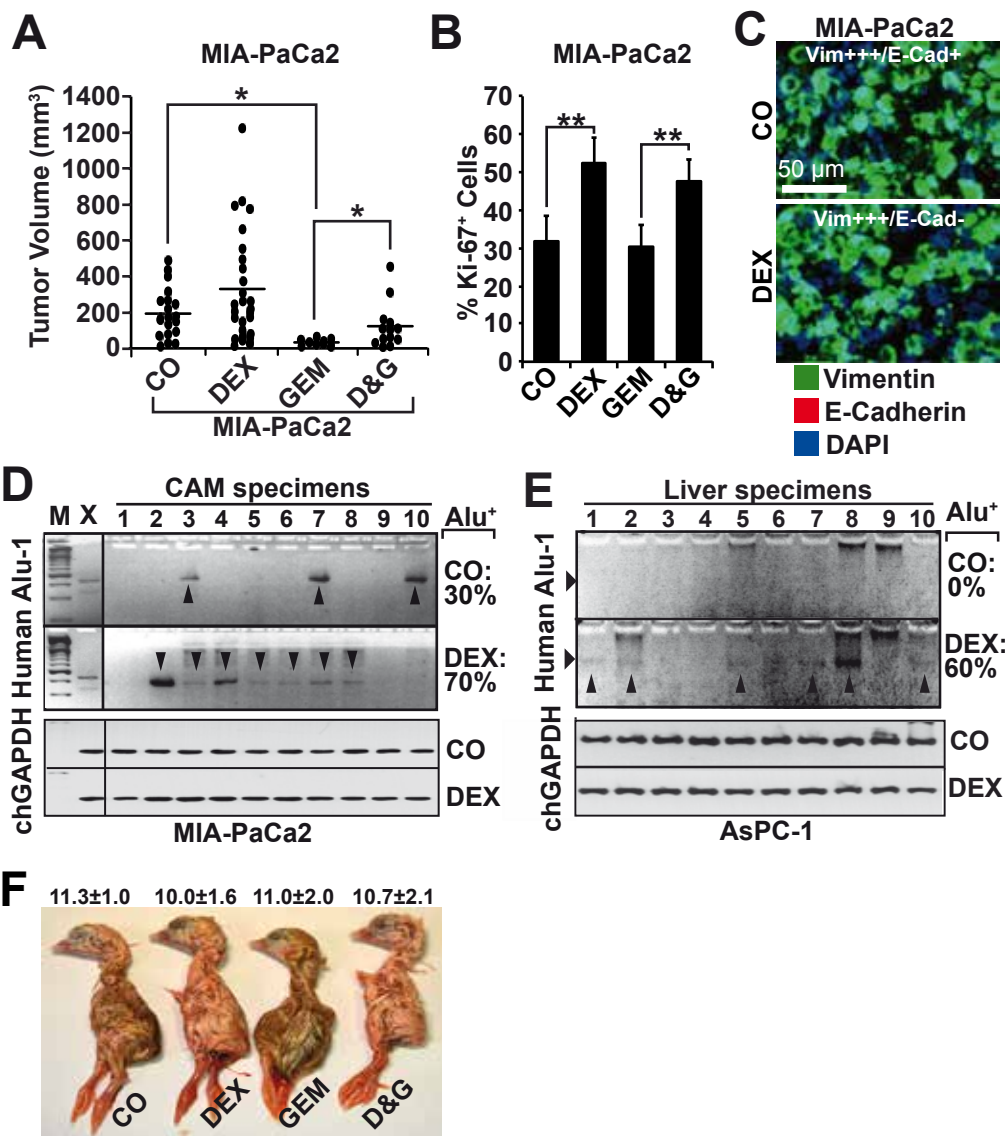

Supplement: Supplementary Figure 1 [file cddis2017455x1.pdf]

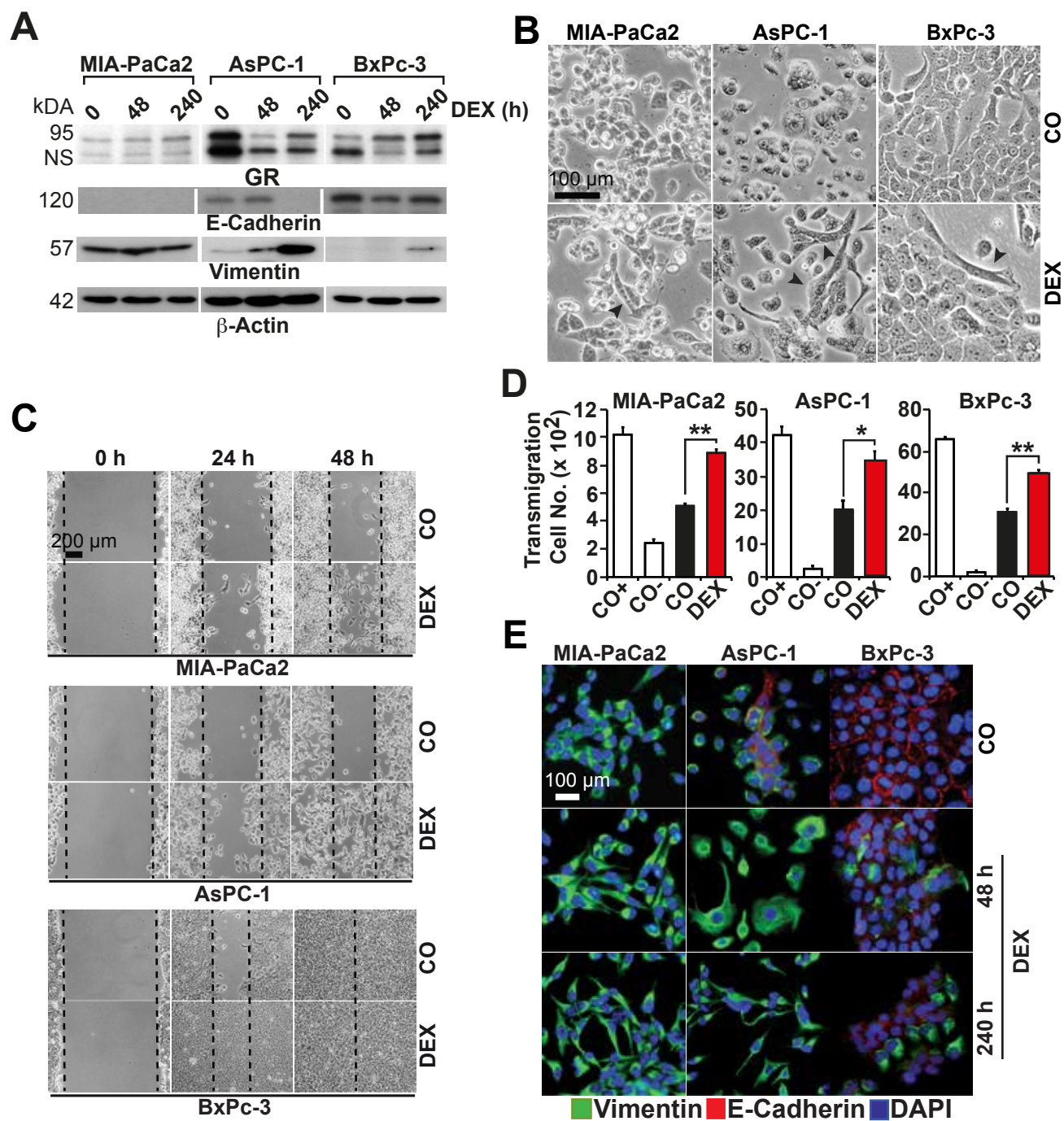

Supplement: Supplementary Figure 2 [file cddis2017455x2.pdf]

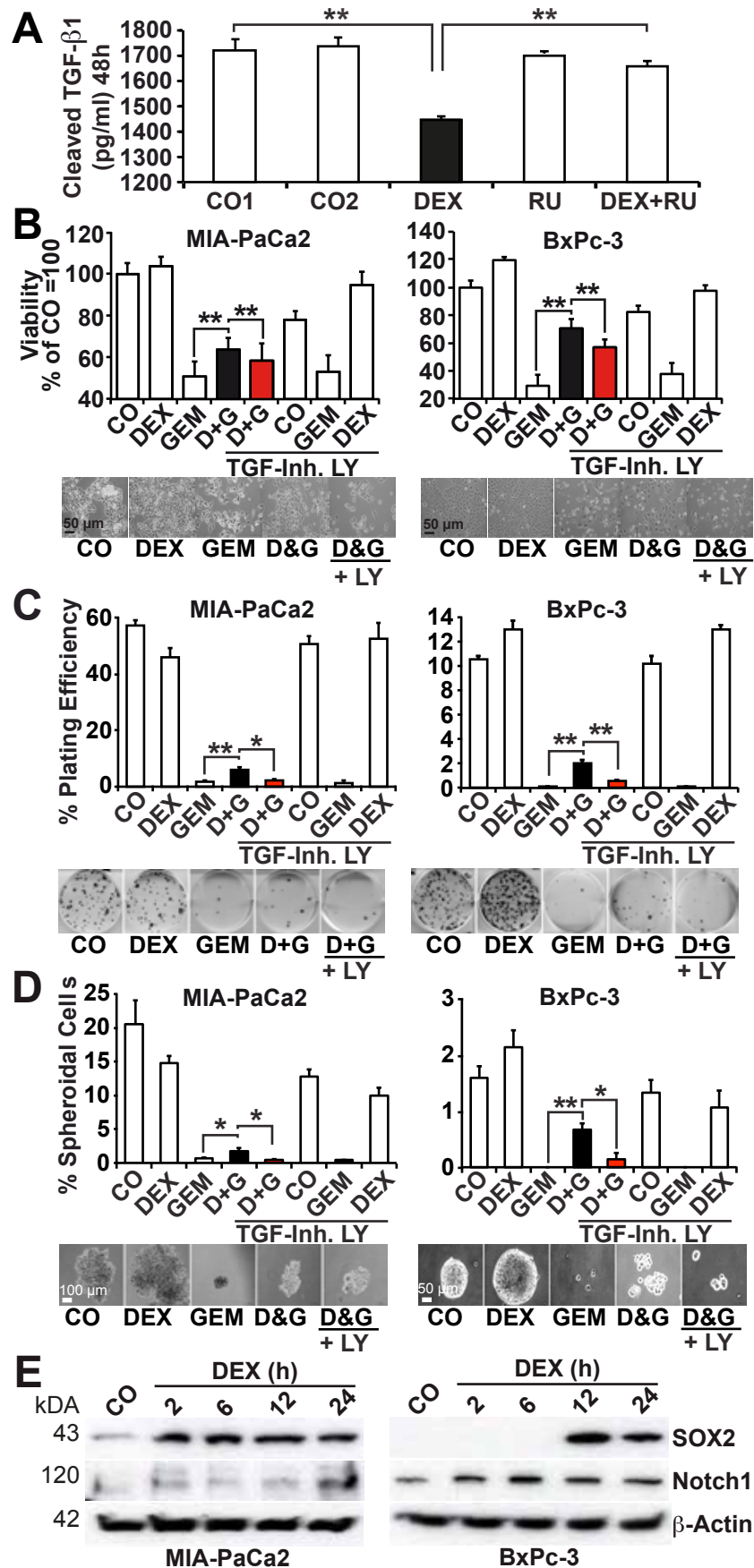

Supplement: Supplementary Figure 3 [file cddis2017455x3.pdf]

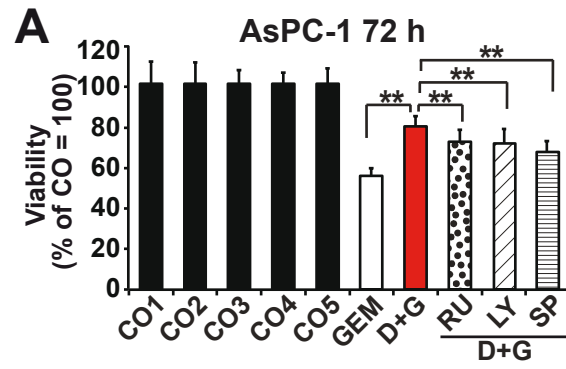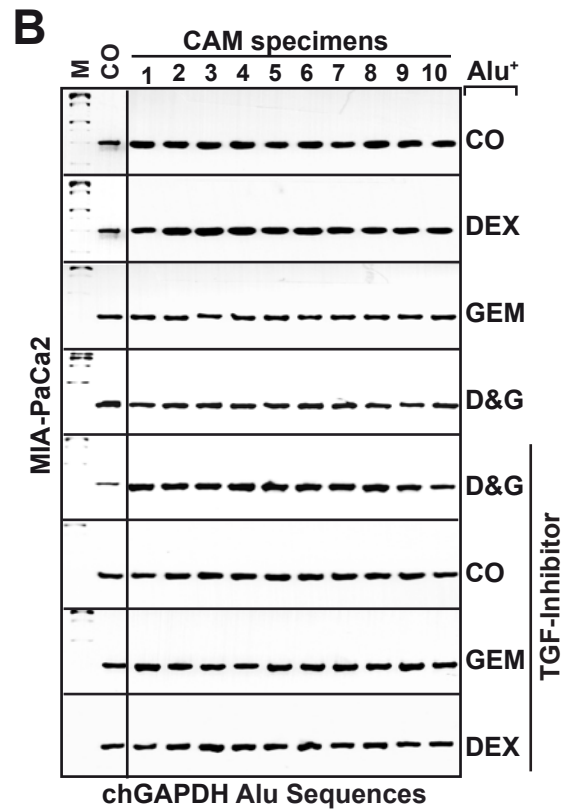

Supplement: Supplementary Figure 4 [file cddis2017455x4.pdf]
