# Supplementary material for: Dexamethasone mediates pancreatic cancer progression by glucocorticoid receptor, TGFβ and JNK/AP-1
Source: Cell Death Dis. 2017 Oct 5;8(10):e3064–. doi: 10.1038/cddis.2017.455 (PMC5680577; doi:10.1038/cddis.2017.455)
Supplement: Supplementary Figure Legends [file cddis2017455x5.docx]

**Supplemental Figure Legends**

**Fig. S1. Dexamethasone induces progression and metastasis in tumor xenografts on eggs. (A-E)** MIA-PaCa2 cells were treated and examined as described in Fig. 2. **(F)** Representative photographs of chick embryos of each treatment group at the end of the experiment, which is shown in Fig. 2A. The weights of the embryos were determined, and they are presented as the mean weights ±SDs.

**Fig. S2. Dexamethasone induces a fibroblast-like phenotype, EMT and migration. (A)** MIA-PaCa2, AsPC-1 and BxPc-3 cells were left untreated (CO) or were cultured in 1 µM dexamethasone (DEX) for 48 h and 240 h. Western blot analysis was performed to detect the expression of E-cadherin and vimentin. β-Actin served as a control for equal conditions. **(B)** The cells were treated as described above and 240 h later morphological changes were documented by microscopy and photography under 400× magnification. **(C)** The cells were treated with 1 µM dexamethasone (DEX) for 24 h, followed by a scratch with the tip of a white pipette through the center of the cell layer. The closure of the wounded region was analyzed 24 and 48 h later by microscopy under 100× magnification. **(D)** The cells were left untreated (CO) or were cultured in 1 µM dexamethasone for 24 h and were seeded in the presence of 2% FCS in the upper wells of transwell plates, whereas the lower wells contained 2% FCS but no cells. Forty-eight hours later, the number of cells that transmigrated to the lower wells was evaluated by describe in materials and methods. The migration of cells to the lower wells with 10% and 1% FCS served as positive (CO^+^) and negative (CO^-^) controls, respectively. *P<0.05, **P<0.01. **(E)** The cells were left untreated (CO) or were cultured in 1 µM dexamethasone (DEX) for 48 or 240 h, and the EMT markers E-cadherin (red) and vimentin^53^ were analyzed by double immunofluorescence staining under 400× magnification. The cell nuclei were stained with DAPI (blue).

**Fig. S3. Dexamethasone enhances TGFβ-dependent self-renewal and stem cell marker expression*.* (A)** The amount of soluble TGFβ1 ligand (pg/ml) in AsPC-1 cell culture supernatant was examined by ELISA assay after 48 h dexamethasone (DEX, 1 µM) treatment as described in Fig.3B. **(B-D)** MIA-PaCa2 and BxPc-3 cells were treated and evaluated as described in Fig. 3, C, D, E. (MIA-PaCa2: 200 cells/well, BxPc-3: 2000 cells/well). **(E)** The cells were treated and evaluated as described in Fig. 3F.

**Fig. S4. Dexamethasone mediates crosstalk of TGFβ-dependent and JNK pathway. (A)** AsPC-1 cells were treated and evaluated 72 h after incubation with gemcitabine as described in Fig. 5A. (B) Internal chick GAPDH PCR control for the Alu-PCR in Fig. 5E and as described in Supplementary Fig. S1F.
